# Artificial intelligence-assisted retinal imaging enables dense pixel sampling from sparse measurements

**DOI:** 10.1038/s44387-025-00038-2

**Published:** 2025-12-09

**Authors:** Vineeta Das, Andrew J. Bower, Nancy Aguilera, Joanne Li, Johnny Tam

**Affiliations:** National Eye Institute, National Institutes of Health, Bethesda, MD USA

**Keywords:** Computational biology and bioinformatics, Neuroscience

## Abstract

High resolution in vivo adaptive optics (AO) imaging has facilitated cellular level assessment of microscopic cone photoreceptors. However, the necessity for dense pixel sampling for good pixel resolution imposes a tradeoff with acquisition speed, leading to motion artifacts and extensive data generation. We introduce an artificial intelligence (AI) assisted imaging framework utilizing residual in residual transformer generative adversarial network (RRTGAN), an AI method that works alongside AO imaging to restore the pixel resolution of sparsely sampled images, circumventing the need for dense sampling. Our results show that RRTGAN can enable data-efficient imaging, restoring high-quality images from just one-fourth of the data and closely matching ground truth images. Cone spacing estimates across four participants aligned well with histology at various retinal locations. These results demonstrate AI assisted imaging’s potential to overcome pixel sampling and imaging speed tradeoff, an important step toward improving the efficiency of routine AO imaging in the clinic.

## Introduction

Advances in biomedical optics have enabled cellular-level visualization of the living human retina^1–4^. For point scanning imaging systems such as confocal microscopes, there is a tradeoff between increasing the density of pixel sampling for improved resolution vs. decreasing pixel sampling for improved acquisition speed. In the case of ophthalmic imaging instruments such as optical coherence tomography (OCT), dense sampling is essential to ensure that the retina is captured at a pixel resolution sufficient for visualizing the microscopic cellular structures. However, dense sampling lengthens the time needed for each frame/volume to be acquired, which in turn exacerbates motion artifacts and image distortions due to continuous eye movements that occur during imaging.

Adaptive optics (AO) is a technology that can be used to sense and correct optical aberrations in the eye, enabling high resolution in vivo visualization of retinal cells^5^. The integration of AO with OCT has facilitated cellular-level, three-dimensional visualization of microscopic retinal cells^6–8^. Higher imaging speed is desirable from adaptive optics optical coherence tomography (AOOCT) systems to more rapidly acquire images and minimize motion artifacts, but this is often at the expense of insufficient pixel sampling depending on the cell being imaged. Although the introduction of high speed swept source lasers such as Fourier domain mode-locked (FDML) lasers^9^ have improved scan rates^10,11^, acquisition speed still remains a challenge. This limitation is exacerbated by the small field of view (FOV) inherent to AO systems (~0.5 mm × ~0.5 mm), necessitating multiple overlapping acquisitions followed by image processing and montaging to visualize larger areas of the retina. Due to the increased pixel sampling required to image smaller cells such as cone photoreceptors, large amounts of data (on the order of terabytes) are typically generated during the acquisition, which can be resource intensive and inefficient. An alternate approach to increasing data efficiency and optimizing the imaging time is to decrease the pixel sampling (i.e. sparsely sample the retina). However, this may lead to sub-optimal image quality resulting from diminished pixel resolution.

In recent years, artificial intelligence (AI) based image enhancement methods have been applied to advanced optical microscopy techniques, including the application of AI for image super resolution^12–15^, reconstruction^16^, denoising^17,18^, and image translation^19–21^. Among the various AI methods, generative adversarial networks (GANs)^22^, which employ two neural networks (generator and discriminator) in an adversarial fashion, have been particularly effective for image enhancement in medical images such as improving image quality^23–25^, denoising OCT images^26,27^, color normalization and virtual staining in histopathology images^28^, and image synthesis in radiology^29^. GANs are typically comprised of a convolutional neural network (CNN)^30^ to represent local characteristics of images. However, this local processing does not take into consideration the structural relationships across larger regions in the images. Increasingly, transformer networks^31^ have received widespread attention in computer vision due to their ability to capture global features in images^15,32–34^. We hypothesize that the structural correlations present in the layered arrangement of the retinal cells can be better represented using the non-local attention mechanism offered by the transformer. Further, incorporating transformer into the GAN framework helps to ensure faithful restoration of the retinal images that maintain structural consistency of the cells. In this paper, we combine the merits of transformer into GAN to develop an AI-assisted imaging framework where AI works alongside imaging to restore pixel sampling from sub-optimally or sparsely sampled AOOCT images.

For AO retinal imaging, sparse sampling is not typically preferred as it could result in the inability to resolve cells. In this paper, we demonstrate that with the help of AI, the pixel sampling of the sparsely sampled images can be restored to the level of the densely sampled images, making sparse sampling a viable option for more efficient imaging. Our AI method effectively restores pixel sampling from only 1/4^th^ of the acquired data while ensuring reliable visualization of the retinal cells. Overall, the AI-assisted AOOCT imaging strategy circumvents the tradeoff between image degradation due to reduced pixel sampling and acquisition time, overcomes speed limitations of imaging systems due to hardware, and opens up the possibility for an alternate strategy to enhance and accelerate imaging for point-scanning optical systems.

## Results

### AI-assisted imaging achieves dense pixel resolution with only a fraction of the data

**Fig. 1:**
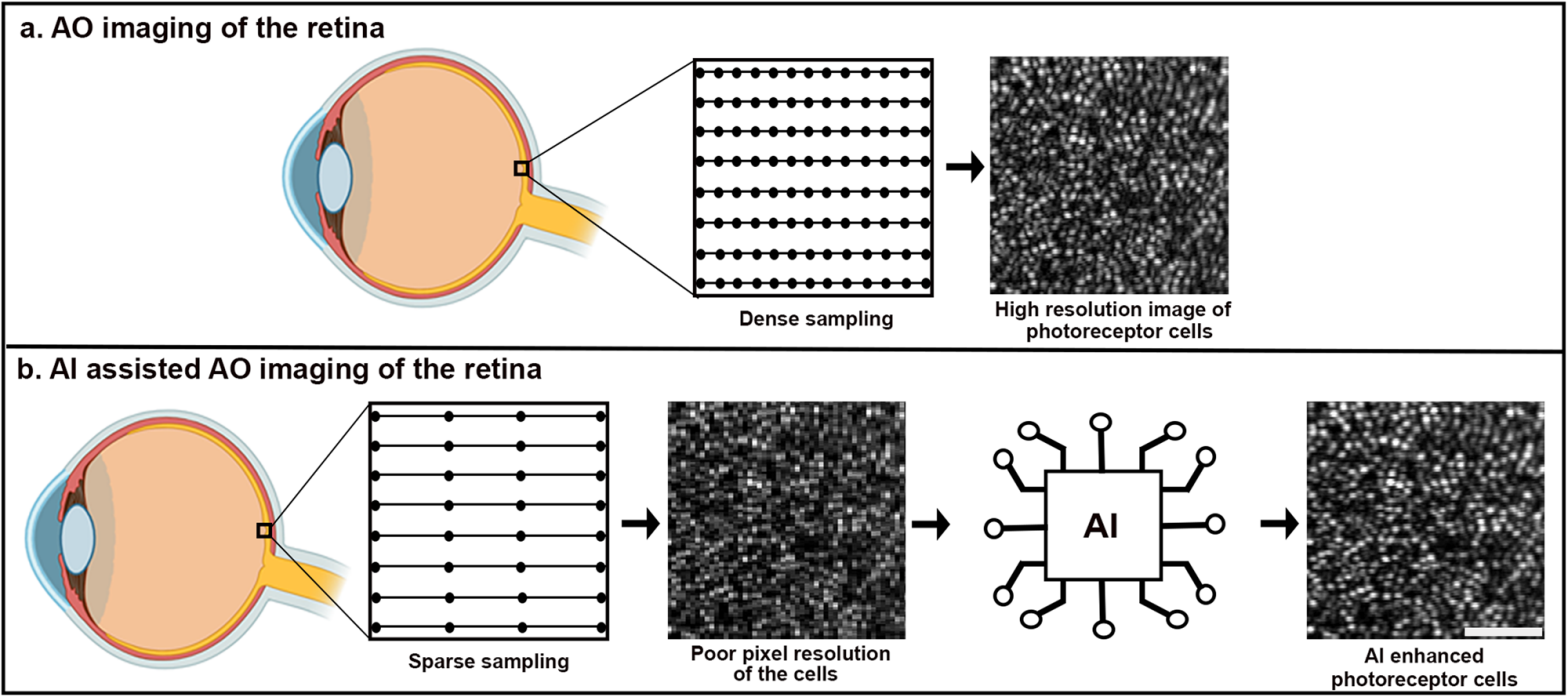
Artificial intelligence (AI) assisted imaging uses sparse sampling to recover cone visualization in the human retina. **a** AOOCT imaging based on densely sampling provides high quality images of the cone photoreceptor cells at the expense of large data sizes and long imaging time. **b** AI-assisted AOOCT imaging sparsely samples the retina and uses AI to restore the pixel resolution of the photoreceptor cells. Incorporating AI into the imaging framework overcomes the tradeoff between pixel sampling and acquisition time, enabling data and time efficient imaging of the retina. The bright dots in the retinal images are individual cone photoreceptor cells. Scale bar: 50 µm.

The overall goal is to integrate AI into the AOOCT imaging framework, where instead of performing dense sampling of the retina to visualize the cells (Fig. 1a), we explore the possibility of performing sparse sampling with only 1/4^th^ of the data required for dense sampling and then use AI to improve the visualization of the poorly sampled cells (Fig. 1b). Our AI algorithm, residual in residual transformer GAN (RRTGAN), incorporates a modified generator based on transformers that are connected in a residual fashion to effectively model the correlations of the different retinal layers captured using the AOOCT to restore the visualization of the individual photoreceptor cells (Supplementary Fig. 1, Supplementary Methods).

**Fig. 2:**
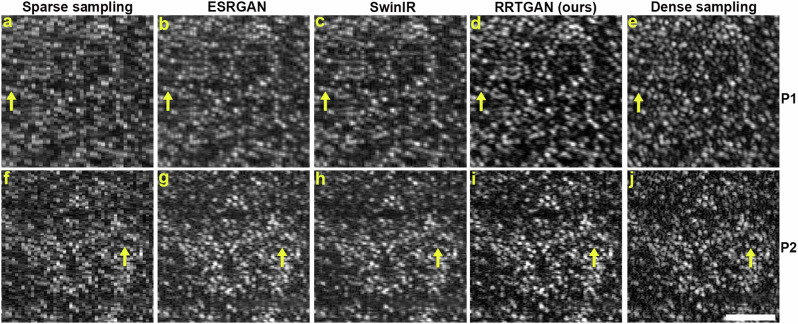
Residual in residual transformer generative adversarial network (RRTGAN) restores the pixel resolution of sparsely sampled images to match dense sampling. **a**, **f** Sparsely sampled images of the cone photoreceptors from two participants (P1 and P2) having a pixelated appearance due to reduced pixel resolution. Pixel resolution of the cones enhanced by AI using (**b**, **g**) enhanced super-resolution generative adversarial network (ESRGAN), **c**, **h** SwinIR, and **d**, **i** RRTGAN (ours). **e**, **j** Ground truth densely sampled images for visual comparison. The bright dots in the images represent the individual cone cells. The yellow arrows show cells that are more distinguishable in RRTGAN compared to the sparsely sampled, ESRGAN, and SwinIR images. Scale bar: 50 µm.

The sparsely sampled images showed a pixelated appearance of the cone photoreceptor cells (Fig. 2a, f). RRTGAN was successful in improving the pixel resolution and enhancing the visualization of cells from sparsely sampled AOOCT volumes (Fig. 2) acquired from study participants (Supplementary Table 1). RRTGAN enhanced and ground truth (densely sampled) cone photoreceptor images showed similarity in cellular structure of the cones (Supplementary Video 1). RRTGAN also performed better than other competitive AI frameworks (ESRGAN and SwinIR) (Fig. 2). Qualitative visual comparison of the images showed clearer and sharper visualization of cones using RRTGAN compared to ESRGAN^14^ and SwinIR^15^. This is likely due to the self-attention mechanism of the dense transformer blocks enabling the learning of non-local features and the skip connections bypassing abundant low frequency information, allowing the generator to focus on learning high frequency information and aiding in accurate restoration of the structure and shape of the cones in the enhanced images.

Quantitative comparison among the methods using objective image quality assessment metrics (peak signal to noise ratio (PSNR), deep image structure and texture similarity (DISTS)^35^, and learned perceptual image patch similarity (LPIPS)^36^) further corroborated our findings on the performance of RRTGAN (Supplementary Fig. 2) across study participants (Supplementary Table 1). For all three metrics, the improvement in RRTGAN performance was statistically significant (*p* < 0.05). RRTGAN images exhibited the highest PSNR indicating better pixel level similarity with the optimally sampled ground truth images. There was an average reduction of 29% in DISTS and 31% in LPIPS for RRTGAN compared to other networks, indicating better perceptual similarity of RRTGAN enhanced and ground truth images. Likewise, RRTGAN also demonstrated the lowest Fréchet Inception Distance (FID)^37^ score of 85.8 compared to ESRGAN (104.4) and SwinIR (127.8) indicating that the enhanced images were more similar to the ground truth images compared to other methods. Overall, our results indicate that RRTGAN outperformed existing AI methods and could successfully enhance the visualization of the cone photoreceptors from sparsely sampled AOOCT.

### AI restores the shape of the cones from sparsely sampled images

**Fig. 3:**
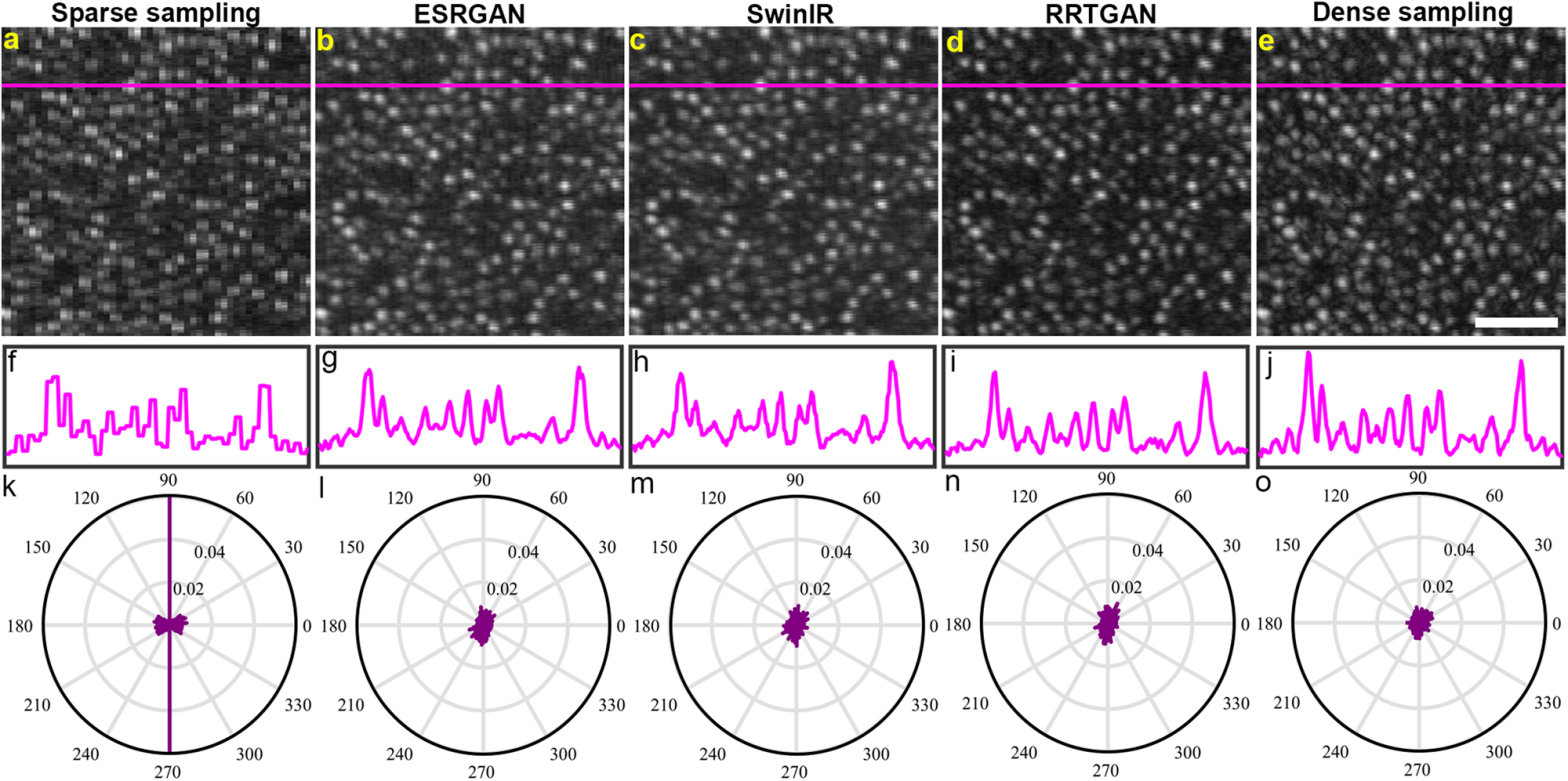
Artificial intelligence (AI) restores shape of the cones from sparsely sampled images. Pixelated appearance of the cones visualized in **a** sparsely sampled images. The cells have smooth edges in **b** enhanced super resolution generative adversarial network (ESRGAN), **c** SwinIR, **d** residual in residual transformer generative adversarial network (RRTGAN), and **e** ground truth images. **f–j** Line intensity profile of the images across the magenta line in (**a**–**e**) shows square peaks for the sparsely sampled image and sharp pointy peaks for the AI-enhanced and ground truth images. **k** Normalized histogram for the sparsely sampled image exhibits edges concentrated in fixed directions corresponding to the sharp edges, reflecting presence of square pixels and hence the pixelated appearance. **l**–**o** The normalized histogram of edges in the AI-enhanced and the ground truth images have edges distributed across all angles indicating the cones in these images no longer have sharp corners from the sparse sampling. Scale bar: 50 µm.

Having demonstrated the efficacy of RRTGAN, we also wanted to evaluate the structural integrity of cones in the enhanced images. Visual inspection of the sparsely sampled images displayed a pixelated appearance (Fig. 3a) of the cones. It was interesting to observe that the individual cone cells did not have pixelation in all AI-enhanced images (Fig. 3b–d). Line intensity profiles through the images showed square peaks at the locations of the cones in the sparsely sampled images (Fig. 3f), confirming that the individual cone cells appear pixilated due to reduced pixel sampling. Sharper peaks corresponding to the cones were observed for the cones in the AI-enhanced and ground truth images (Fig. 3g–j).

Using normalized histograms to measure the directionality of the edges (Methods, Edge Directionality) in the sparsely sampled images also revealed a higher concentration at the vertical directions, further confirming the presence of sharp edges (Fig. 3k). On the contrary, the AI enhanced and the densely sampled ground truth images had edges uniformly distributed across different angles, corroborating the smoother appearance of the cones seen in the AI enhanced images (Fig. 3l–o). Computing the variance in the edge direction from the normalized histogram showed less variance for the AI methods compared to the sparsely sampled images (Supplementary Table 2). These results reinforce our observation demonstrating the potential of AI for removing sharp edges and restoring the smooth shape of the microscopic photoreceptor cells.

### RRTGAN preserves cell spacing and contrast comparable to densely sampled images

**Fig. 4:**
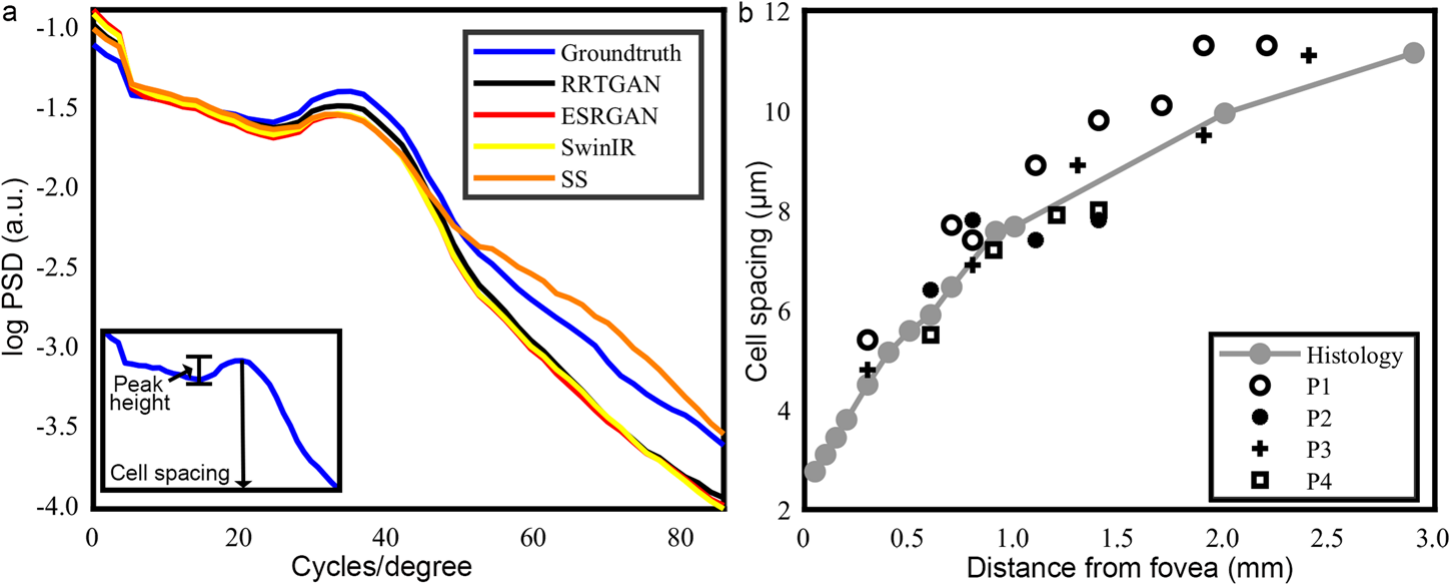
Cone cell spacing of the artificial intelligence (AI) enhanced images are comparable to the ground truth densely sampled images. **a** Circumferentially averaged power spectral density (PSD) of the sparsely sampled (SS), AI-enhanced and ground truth images of participant P3 imaged at 1.3 mm temporal to the fovea. The visible peak corresponding to cone cell spacing is observed in all the images. The inset shows a zoomed version of the peak with the vertical black line indicating the location of the fundamental spatial frequency associated with the cells. The height of the peak indicates the cellular contrast. **b** Comparison of cone spacing estimated from residual in residual transformer generative adversarial network (RRTGAN) enhanced images with previously published histology data^39^. Symbols in black indicate cell spacing estimated from RRTGAN-enhanced images for four participants (P1, P2, P3, and P4) at different retinal locations. Cone spacing replotted from histology^39^ shown in gray.

In addition to demonstrating the efficacy of RRTGAN in improving both the visualization and circular shape of cones, we also objectively evaluated how well RRTGAN preserves the spatial distribution of the cones in the images. Examination of the radially averaged power spectral density showed a peak indicating the fundamental spatial frequency (representative of cell spacing) associated with the spatial arrangement of the cones in the images^38^. The peaks for the ground truth densely sampled and AI images were consistent (Fig. 4a and Supplementary Table 3), indicating high fidelity of the RRTGAN enhancement.

Furthermore, the height of the local peak of the radially averaged power spectral density provided an objective way to quantify the cellular contrast. Compared to the sparsely sampled cones, ESRGAN and SwinIR enhanced images, the RRTGAN enhanced images have the highest peak height, indicating the best cellular contrast and visualization of the cells (Table 1). The peak height of RRTGAN and the ground truth images are remarkably close, which agrees with our earlier results, indicating the improved visualization offered by RRTGAN. These results demonstrate RRTGAN’s effectiveness in boosting cellular contrast in addition to providing structural and perceptual similarity.

**Table 1:** Comparison of cellular contrast of the different methods

Image	Peak height (a. u.)
Sparse sampling	0.06 ± 0.03
ESRGAN	0.09 ± 0.03
SwinIR	0.09 ± 0.03
RRTGAN (ours)	0.12 ± 0.02
Dense sampling	0.11 ± 0.06

The cell spacing estimated using the circumferentially averaged power spectral density for RRTGAN-enhanced images across all participants were within the expected ranges when compared with previously published normative data (Fig. 4b), confirming that RRTGAN did not by create or delete additional cells. These results demonstrate the possibility of using AI to restore pixel sampling from insufficiently sampled data, which could enable AOOCT to more efficiently visualize cells from different retinal locations in the living human eye.

### AI-assisted imaging generalizes across the retina

**Fig. 5:**
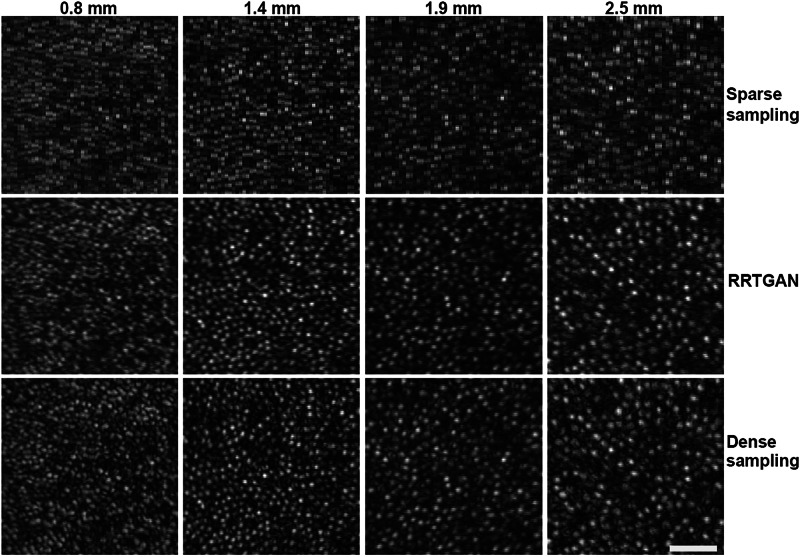
Residual in residual transformer generative adversarial network (RRTGAN) and the ground truth densely sampled images have similar appearance of the cones for experimental data at different retinal locations. Sparsely sampled, RRTGAN enhanced, and ground truth images are similar across all retinal locations (0.8, 1.4, 1.9, and 2.5 mm temporal to the fovea). Images are shown from participant P1 (not used for model training). RRTGAN is able to effectively enhance images with varying cell size and spacing across different retinal locations and restores images to resemble cells seen in ground truth images. Scale bar: 50 µm.

Cone distribution across the retina varies significantly, with the highest density concentrated near the fovea, gradually decreasing as the distance from the fovea increases^39^. Additionally, cone cells are also much smaller near the fovea and progressively increase in size with increasing distance from fovea. Having trained and validated the performance of RRTGAN on images collected approximately 1 mm from the fovea, we now evaluate its effectiveness on images from retinal locations that were never seen by RRTGAN during training. We tested the performance of RRTGAN at retinal locations 0.8 mm, 1.4 mm, 1.8 mm, and 2.5 mm temporal to the fovea, and found that RRTGAN successfully recovered the cone cellular structures at all the four locations (Fig. 5). Objective quantification further confirmed the similarity of RRTGAN-enhanced and the ground-truth images (Supplementary Table 4). This result demonstrates that RRTGAN can generalize across different imaging locations and can faithfully recover the cellular structures for never seen cells of different sizes.

**Fig. 6:**
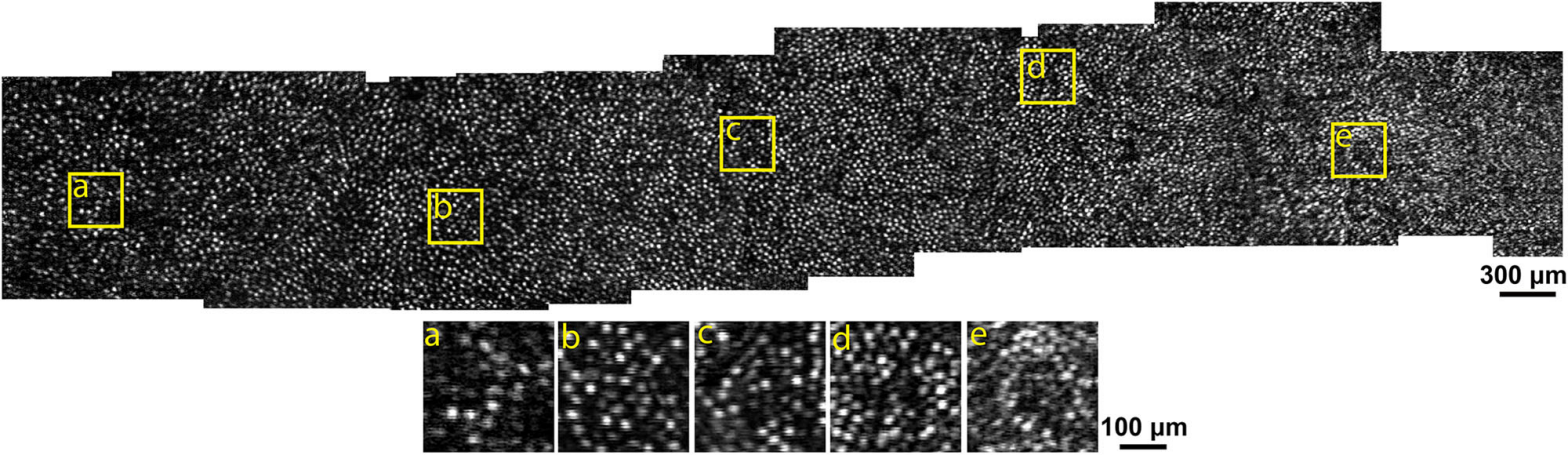
Residual in residual transformer generative adversarial network (RRTGAN) enables efficient large-scale visualization of cone photoreceptors across the retina. The image shows the visualization of the cone photoreceptors using the RRTGAN-enhanced images. The montage is constructed by stitching together more than 20 overlapping images from the right eye of participant P1. The yellow squares (**a**–**e**) indicate regions that have been further zoomed for better visualization at retinal locations **a** 2.6, **b** 2.1, **c** 1.7, **d** 1.2, and **e** 0.8 mm temporal to the fovea.

Having demonstrated the generalizability of RRTGAN across different retinal locations featuring a range of cell sizes, we also verified the consistency of the cells by imaging two contiguous retinal locations with overlapping regions. Consistency of cells across regions is essential for stitching together individual images with smaller FOV in order to investigate changes to the retina occurring over wider retinal regions. Two RRTGAN-enhanced images obtained from contiguous locations with ~50% overlap showed that the cells in the overlapping areas are consistent in both images, verifying that the AI-enhanced images preserve the spatial locations of the cones (Supplementary Video 2). By extending this approach across 23 overlapping retinal locations, this feat allowed us to apply RRTGAN to a much larger retinal region spanning across 3 mm temporal to the fovea (Fig. 6). This shows that the AI-enhanced images can be successfully used to build large montages to visualize larger areas of the retina.

**Fig. 7:**
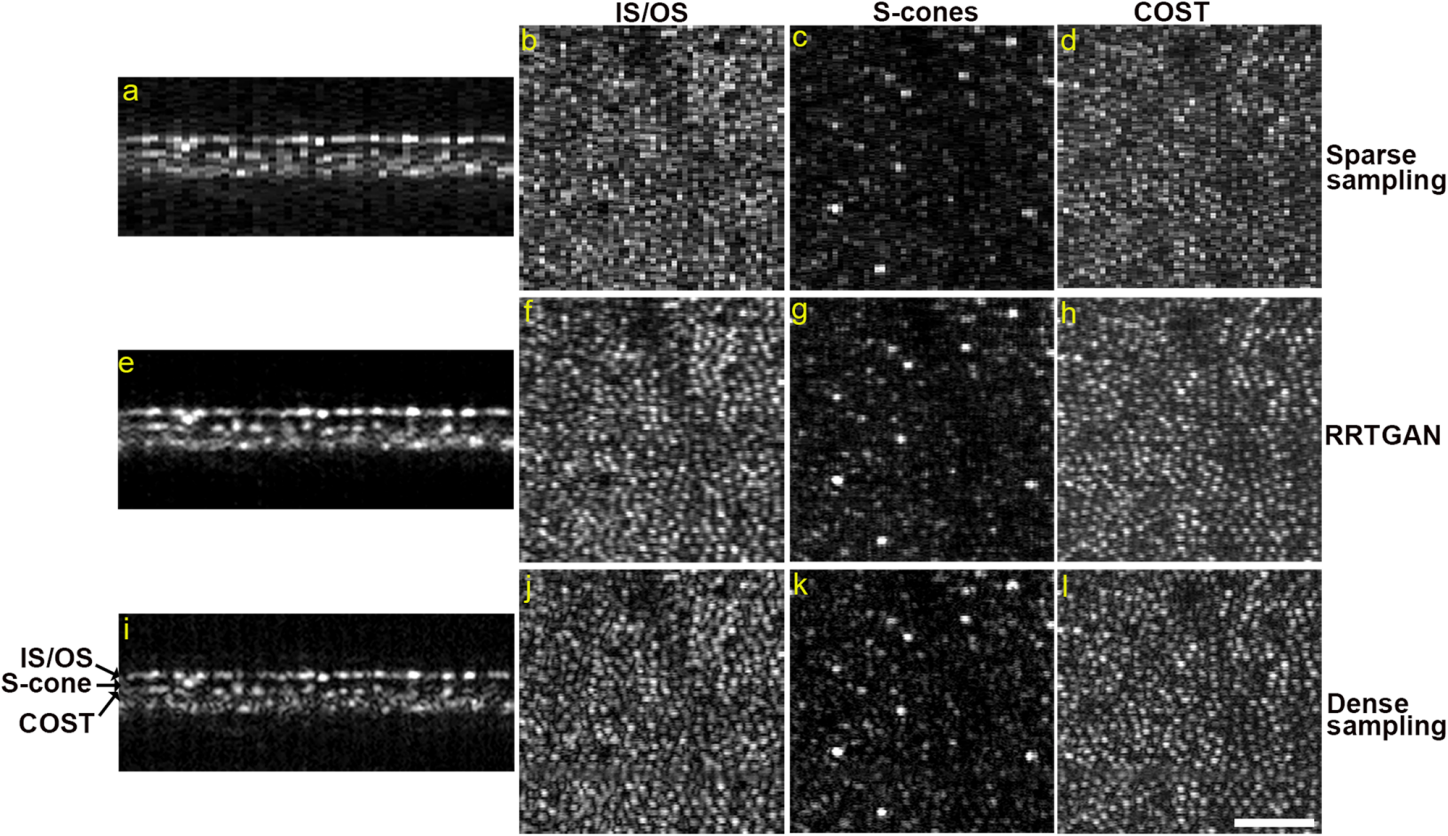
Residual in residual transformer generative adversarial network (RRTGAN) restores the 3D structure of cones. **a**, **e**, **i** Cross-sectional adaptive optics optical coherence tomography (AOOCT) B-scans of the sparsely sampled, RRTGAN-enhanced, and ground truth densely sampled retina of participant P4 at 1.2 mm temporal to the fovea. **b** Top-down view of the inner segment/outer segment (IS/OS) junction that appear as bright dots are hard to distinguish visually in the sparsely sampled images and have better visualization in (**f**) RRTGAN enhanced and (**j**) ground truth densely sampled images. Similar observations are seen for (**c**, **g**, **k**) S-cones and (**d**, **h**, **l**) cone outer segment tips. The arrows in (**i**) highlight the IS/OS junction, S-cones, and COST layers of the retina in the B-scan. Scale bar: 50 µm.

Finally, thus far, we showed the RRTGAN effectively enhanced the cone photoreceptor layer (also referred to as the ellipsoid zone layer or the inner segment/outer segment (IS/OS) junction), which is only a portion of the overall 3D AOOCT volume. Since RRTGAN operates on the cross-sectional 2D AOOCT images (B-scans), it naturally operates across all of the neighboring layers that are in focus on the outer retinal AOOCT volumes. We found that RRTGAN was also successful in restoring the S cone layer^40^ (cones sensitive to short wavelength light) as well as the cone outer segment tips (COST) layer of the retina (Fig. 7). In addition to enhancing the cone photoreceptors, since RRTGAN enhances data across the entire AOOCT B-scan, it was also effective in restoring the visualization of neighboring layers (Supplementary Fig. 3). This result shows an advantage of the RRTGAN training using cross-sectional 2D AOOCT images (i.e. OCT B-scans), extending its benefits across multiple 3D layers that can be reconstructed to obtain multiple rotated 2D views of the tissue (i.e. OCT en face view).

## Discussion

We demonstrated that RRTGAN successfully restored the pixel sampling from sparsely sampled AOOCT images. The resulting RRTGAN-enhanced images enabled both the visualization of cone photoreceptor cells across the retina as well as the quantification of structural metrics such as cell-to-cell spacing despite using only one fourth of the imaging data. The enhanced images also successfully restored the 3D cone structure and enabled efficient visualization of the IS/OS junction and COST layers. Because RRTGAN is trained on cross-sectional B-scans rather than en face photoreceptor images, it also enables improved visualization of neighboring layers, including the retinal pigment epithelium (RPE) and choroidal vessels. This approach is also more data efficient as it can be applied to 2D slices and generalized across a 3D volume, enabling more rapid cellular-scale 3D imaging of the eye in a clinical setting.

The success of RRTGAN can be attributed to the self-attention mechanism of transformers in effectively learning global correlations from the retinal layers of the AOOCT images together with the ability of the GAN to restore cellular structures. The improvement of RRTGAN over the CNN based ESRGAN^14^ indicated that the restoration benefitted from non-local self-similarity modeling offered by the transformers. RRTGAN also generated superior results compared to the state-of-the-art SwinIR framework. This could be attributed to the residual in residual connections in RRTGAN that aided in improving the flow of gradients through the network. In terms of computational complexity, RRTGAN also has fewer parameters than ESRGAN (Supplementary Table 5). It was interesting that although trained on 2D cross-sectional image slices instead of 3D AOOCT volumes, RRTGAN was still able to preserve the 3D structure of the cells for visualization. This further illustrates the consistency of the network in independently enhancing neighboring B scans (one at a time) in a manner that still results in a seamless transition when viewing cells that are split across neighboring B scans.

Our validation confirmed that the RRTGAN-enhanced images accurately preserved the cellular structure, which is crucial for assessing retinal health, particularly for performing morphometric measurements from the cells. This integration of AI into the AOOCT image acquisition process represents an approach, diverging from the traditional post-acquisition applications of AI. RRTGAN working alongside AOOCT imaging guarantees data efficiency as the images with 75% fewer samples can be restored to match the ones acquired with 100% sampling. Our AOOCT system can quickly generate terabytes of data across a typical imaging session (3D volumes from 20 to 25 locations per eye, with ~50 GB for 85 volumes acquired at each location). With RRTGAN, the requirement of dense sampling can be relaxed and thereby substantially reducing the data size. Further, the time saving achieved by acquiring less data also allows imaging at more retinal locations in the same amount of imaging time, particularly advantageous in a clinical setting where reducing the time spent per patient is important. This also results in substantial savings in the computational time needed to post-process AOOCT volumes to correct for eye motion^27^. Given the success of RRTGAN in enhancing the visualization of cone cells, we anticipate that such an approach can potentially be translated to other speed-constrained imaging applications such as AOOCT angiography^41^, which requires multiple scans of the same location in quick succession.

In conclusion, we introduced an AI-based RRTGAN framework to improve the visualization of cells from sparsely sampled, rapidly acquired, low pixel resolution images. By adopting an AI-assisted imaging strategy^24,27^, we demonstrated the capability of AI not only in improving the visualization of cells and reducing the time needed to acquire images, but also providing an opportunity to image at higher speeds as hardware solutions improve. Addressing both the technical limitations of AOOCT systems and the practical burden on patients and clinicians is essential for broader clinical adoption. Our findings highlight the transformative potential of AI in retinal imaging and contribute meaningfully to the first step towards translation of AOOCT into routine ophthalmic practice.

## Methods

### Adaptive optics optical coherence tomography instrument

A custom built swept-source AOOCT instrument (Supplementary Fig. 4) was used, based on an FDML light source^42^ with central wavelength of 1060 nm and bandwidth of 77 nm (NG-FDML-1060-750-8B-FA, Optores, Munich, Germany) combined with a wavefront sensor (WFS) beacon at 940 nm (SLD-mCS-481-HP2-PM, Superlum, Carrigtwohill, Co. Cork, Ireland). Light was raster scanned in the horizontal direction using a 3.3 kHz resonant scanner (SC-30, Electro-Optical Products Corp, Fresh Meadows, NY, USA) and in the vertical direction using a tip-tilt scanner (S-334, PI-USA, Auburn, MA, USA). Wavefront correction was performed using a deformable mirror (DM97, Alpao, Montbonnot, France).

### Adaptive optics optical coherence tomography imaging of human participants

Participants with no history or signs of ocular disease were recruited for this study. All participants underwent a comprehensive ophthalmic assessment. In total, four eyes from four healthy participants (age: 42.8 ± 13.0 years) from the National Eye Institute Eye Clinic (National Institutes of Health, Bethesda, Maryland, USA) were imaged using the custom-built AOOCT retinal imaging system. Eyes were dilated with 2.5% phenylephrine hydrochloride (Akorn Inc.) and 1% tropicamide (Sandoz, A Novartis Division). This study was approved by the Institutional Review Board of the National Institutes of Health (NCT02317328). Research procedures adhered to the tenets of the Declaration of Helsinki. Written, informed consent was obtained from all participants after the nature of the research and possible consequences of the study were explained.

### Data for training and validating the artificial intelligence methods

AOOCT volumes from four eyes of four participants were acquired at 12.7 volumes per second with 512 × 512 A-scan sampling across 1.5 degrees field of view. Volumes were acquired from 5 to 23 retinal locations ranging from 0 to 3 mm temporal to the fovea. At each location, 45–85 volumes were acquired. Following image acquisition, the volumes were digitally flattened to correct for axial eye motion based on the outer retinal layers. The volumes were cropped at the edges to remove image portions with scanner distortion artifacts near the turnaround points of the scanner, resulting in final size of 512 × 400 × 378 pixels.

To train the AI methods, AOOCT B-scans were extracted from volumes of three participants at locations 0.8–1.2 mm. The region of interest (40 × 400 pixels) was cropped from the B-scans to form densely sampled ground truth images. These images were then down-sampled by a factor of 4 in the lateral direction to obtain sparsely sampled images (40 × 100 pixels). More than 10,000 densely/sparsely sampled image pairs were used for training.

To test the model, a leave one subject out based validation scheme was adopted. During testing, each B-scan of the volume is enhanced using AI (80 × 100 pixels; note the cropping is different than during training). The AI enhanced volumes were then registered to correct for eye motion artifacts and averaged to visualize the cones. The proposed RRTGAN model was also tested on a participant that had never been seen during the training process to validate the generalizability of the method.

### Validation metrics

#### Objective image quality assessment

Four image quality assessment metrics were used in this study: peak signal to noise ratio (PSNR), deep image structure and texture similarity (DISTS)^35^, learned perceptual image patch similarity (LPIPS)^36^, and Fréchet Inception Distance (FID)^37^. PSNR is a simple and widely used measure of the quality of the enhanced and the corresponding ground truth images. It computes the distortion by measuring pixel differences between the enhanced and the ground truth images, with higher values indicating better quality. DISTS and LPIPS extract deep features to assess the image quality of the AI-enhanced images. DISTS measures the textural and structural similarity between enhanced and ground truth images using deep features extracted from five layers of the VGG16 network. LPIPS quantifies human perceptual similarity between enhanced and ground truth images by computing the L2 distance between unit-normalized and scaled features extracted from multiple layers of the VGG network. FID evaluates the AI frameworks by assessing the quality of the generated images compared to the ground truths. A lower FID score guarantees better quality of the generated images.

#### Cell spacing and contrast measurement

Cell spacing and contrast was quantified to assess the efficacy of RRTGAN for cone photoreceptor enhancement. Cell spacing was estimated using the circumferentially averaged power spectrum^38^ of each image region of interest. The peak spatial frequency of the spectrum (i.e., the cone photoreceptor fundamental frequency) is an estimate of cell spacing. To convert from pixels to µm, a paraxial ray trace on a three-surfaced simplified model eye^43^ is used after replacing the axial length, corneal curvature, and anterior chamber depth with measurements of these values obtained from each participant (IOL Master, Carl Zeiss Meditec)^44^. Cellular contrast was approximated by measuring the height of the local peak in the circumferentially averaged power spectrum.

#### Edge directionality

Edge directionality of the images was measured to assess the degree of pixelation in the images. It was calculated by first obtaining the edges of the individual cone cells. At the location of the edges, their direction was computed as ${\arctan }\frac{{G}_{y}}{{G}_{x}}$, where ${G}_{y}$ and ${G}_{x}$ were the vertical and horizontal edges, respectively. Histograms of the directions were used to visualize the distribution of edge directionalities. Higher magnitudes at 90 and 270 degrees in the histogram were indicative of presence of sharp edges and are useful to detect when cones exhibited a pixelated appearance. Uniformly distributed edges across all angles indicated smooth edges of the cones.

### Statistical analysis

Statistical analysis was performed using a one-tailed paired Student’s *t* test to determine if there was statistically significant improvement in cell visualization between the sparsely sampled and the AI enhanced images. The test was applied to the objective image quality metrics (peak signal to noise ratio (PSNR), deep image structure and texture similarity (DISTS), and learned perceptual image patch similarity (LPIPS)) for both image sets for all four participants (*N* = *4*).

## Supplementary information

spatial_enhancement_SI_revised_v2_clean

Supplementary Video 1

Supplementary Video 2

## Data Availability

All data is included in the manuscript and/or supporting information.

## Code availability

The PyTorch implementation of RRTGAN is publicly available at https://github.com/NIH-NEI/RRTGAN.
