# Supplementary material for: Artificial intelligence-assisted retinal imaging enables dense pixel sampling from sparse measurements
Source: NPJ Artif Intell. 2025 Dec 9;1(1):48. doi: 10.1038/s44387-025-00038-2 (PMC12689425; doi:10.1038/s44387-025-00038-2)
Supplement: Supplementary file 1 — spatial_enhancement_SI_revised_v2_clean [file 44387_2025_38_MOESM1_ESM.pdf]

Supplementary Information

Artificial Intelligence Assisted Retinal Imaging Enables Dense Pixel Sampling from Sparse Measurements

Vineeta Das, Andrew J Bower, Nancy Aguilera, Joanne Li, and Johnny Tam
National Eye Institute, National Institutes of Health, Bethesda, MD, 20892, USA

Supplementary Methods

Residual in residual transformer generative adversarial network (RRTGAN) architecture

The generative adversarial network (GAN) introduced in [1] has been very successful in generating realistic looking images and has proven useful for a plethora of image translation tasks such as super-resolution [2, 3], denoising [4, 5] and image reconstruction [6, 7]. GAN uses two convolutional neural networks (CNN), generator and discriminator, that work simultaneously in an adversarial fashion to create realistic images. In this work, we have modified the generator of GAN by introducing the transformer neural network to better capture the non-local self-similar features and global dependencies to further improve the perceptual quality of the generated images.

The generator of RRTGAN takes the sparsely sampled AOOCT cross sectional images (B-scans) as input and learns how to enhance it to match the ground truth densely sampled images (**Supplementary Fig. 1**). To achieve this, the generator first utilizes a convolutional layer to extract low-level spatial features from the input images. These feature maps are then provided to a series of five residual transformer groups that use a complex arrangement of self-attention layers to extract high frequency edge details and non-local features. The residual transformer group has dense residual blocks as its core unit. The dense residual blocks employ residual connections at different levels among its building blocks which are the window based self-attention block (WSAB) layers. With the long and short residual skip connections in the framework, the generator can transmit low level features to the deeper layers, which can then allow the deeper layers to focus on learning the high frequency details, improving the overall image quality.

The emergence of transformers provides an opportunity to model non-local self-similarity and long-range dependencies in images, but the computational cost of the global transformer is quadratic to the spatial size of the input image. This burden is non-trivial. Hence, inspired by [8], we adopt the window-based multi-head attention (W-MSA) in WSAB. In W-MSA, the input feature map $X_{in} \epsilon \mathbb{R}^{H \times W \times C}$ is partitioned into non-overlapping $M \times M$ local windows. Multi-head self-attention is then computed separately for each window. Given the feature map X_{in}, the feature of the l-th window is denoted as $X_{in}^{l} \in \mathbb{R}^{M \times M \times C}$, where $l \, \epsilon \, \{1,2, \dots, \frac{HW}{M^2}\}$. The windowed features are flattened and transformed into $X_{in}^{l} \epsilon \, \mathbb{R}^{M^2 \times C}$ to compute the multi-head self-attention. The query (Q^l), key (K^l), and value (V^l) matrices are computed as

$$Q^l = X^l W_Q, \quad K^l = X^l W_K, \quad V^l = X^l W_V \tag{1}$$

where W_Q, W_K and W_V are learnable parameters. Q^l, K^l, and V^l are split into k heads along the channel direction: $Q^l = [Q_1^l, Q_2^l, \dots, Q_k^l]$, $K^l = [K_1^l, K_2^l, \dots, K_k^l]$, and $V^l = [V_1^l, V_2^l, \dots, V_k^l]$. The self-attention for each head *j* is formulated as

$$SA(Q_j^l, K_j^l, V_j^l) = Softmax\left({Q_j^l K_j^{l^T}} \Big/ {\sqrt{d_k}} \right) V_j^l, \text{ where } d_k = \frac{C}{k} \tag{2}$$

The self-attention of all windows is concatenated as

$$X_o^l = Concat\left(SA\left(Q_j^l, K_j^l, V_j^l\right)\right) W_o + B \tag{3}$$

where B represents the position embedding and $W_o \epsilon \, \mathbb{R}^{C \times C}$ are learnable parameters. X_o^l is reshaped to $\mathbb{R}^{M \times M \times C}$. Finally, all the window features are merged to obtain the output feature map $X_{out} = \{X_{out}^1, X_{out}^2, \dots, X_{out}^N\}$, $X_{out} \, \epsilon \, \mathbb{R}^{H \times W \times C}$. The W-MSA layer is preceded and followed by layer normalization

followed by three convolutional layers. In this work, the size of the input training images is 40 × 100 pixels, the size of local window 5 × 5, and 8 heads are used for the multi-head attention.

The output from the last residual group is summarized using a convolutional layer to bring the convolution's inherent inductive bias to the network. An interpolation operation is used to restore the feature maps to the original size. In the end, a convolutional layer with tanh activation is used to aggregate the feature maps and generate the output image. The discriminator of RRTGAN consists of a series of five convolutional layers with leakyReLu (LReLu) activation followed by a global average pooling layer and a linear dense layer. Details on the training hyper-parameters are provided in **Supplementary Table 5**.

Objective loss function

The choice of objective loss function is essential for guiding the generator and discriminator networks during training. These loss functions measure how well each network is performing its respective task and help adjust their parameters through back propagation. We have used three loss functions to train the network.

Content loss, L_c *:*This term measures how well the images enhanced by the generator preserves the content of the ground truth images by measuring the pixel differences between them as $L_c = ||I_{en} - I_{gt}||_1$, where I_{en} and I_{gt} are the generator enhanced and the ground truth images respectively.

Perceptual loss, L_p*:* This term compares high-level features such as texture and shape of the cells in the enhanced and the ground truth images. To measure if the images are perceptually similar, features from the convolutional layer before activation of the 3rd block of the pre-trained VGG19 are extracted and compared using the squared L2 norm as $L_p = ||\phi(I_{en}) - \phi(I_{gt})||_2^2$, where $\phi(.)$ is the feature map from the 3rd conv block of VGG19 network.

Adversarial loss: This term is the core mechanism behind GAN. It drives the interaction between the generator and discriminator, enabling the generator to create realistic images by learning from the feedback of the discriminator. Adversarial loss measures how well the generator is fooling the discriminator and how accurately the discriminator can distinguish between the ground truth and the generator created images. We use the relativistic adversarial loss [2] for the discriminator where instead of estimating the probability that the input image is real, the relativistic discriminator loss tries to predict the probability that a real image is relatively more realistic than the generated one.

The adversarial loss for the generator (L_g) is

$$L_g = -\mathbb{E}_{x_{gt}}[\log(1 - D(x_{gt}, x_{en}))] - \mathbb{E}_{x_{en}}[\log\left(D(x_{en}, x_{gt})\right)] \quad (4)$$

Hence, the total generator loss is $L_c + \lambda L_p + \mu L_g$, where $\lambda = 0.05$ and $\mu = 0.5$ are coefficients to balance different loss terms.

The discriminator adversarial loss (L_d) is given as

$$L_D = -\mathbb{E}_{x_{gt}}[\log(D(x_{gt}, x_{en}))] - \mathbb{E}_{x_{en}}[\log\left(1 - D(x_{en}, x_{gt})\right)] \quad (5)$$

where $D(x_{gt}, x_{en}) = \sigma(C(x_{gt}) - \mathbb{E}_{x_{en}}[C(x_{en})]$, $C(.)$ is the output from the discriminator, and $\mathbb{E}_{x_{en}}$ represents the operation of taking the average of all the generated data in the mini-batch.

Other networks

Enhanced super resolution generative adversarial network (ESRGAN) [2]***:*** ESRGAN is inspired by SRGAN [3] and has been improved to enhance the visual quality of the generated images. ESRGAN introduced the residual-in-residual dense block (RRDB) as their basic building block of the generator network. Each of the RRDBs have a deeper and more complex connections of convolutional layers to increase network capacity to boost performance. A relativistic discriminator was introduced that tries to predict the probability that a real (ground truth) image is relatively more realistic than a fake (generated) one. Five RRDBs are used in the ESRGAN used in the comparison.

SwinIR: The SWINIR [9] network consists of three modules: shallow feature extraction that uses CNN to extract spatial features, followed by the deep feature extraction module that consists of residual swin transformer blocks and a convolutional layer. Each of the swin transformer blocks have swin transformer layers that rely on local attention and shifted window mechanism where the input feature maps are partitioned into non-overlapping local windows and multi-head self-attention is computed separately on each of the windows. Next a multi-layer perceptron that has two fully connected layers with GELU non-linearity between then used for feature transformation. More details of SwinIR can be found in [9].

Supplementary Figures

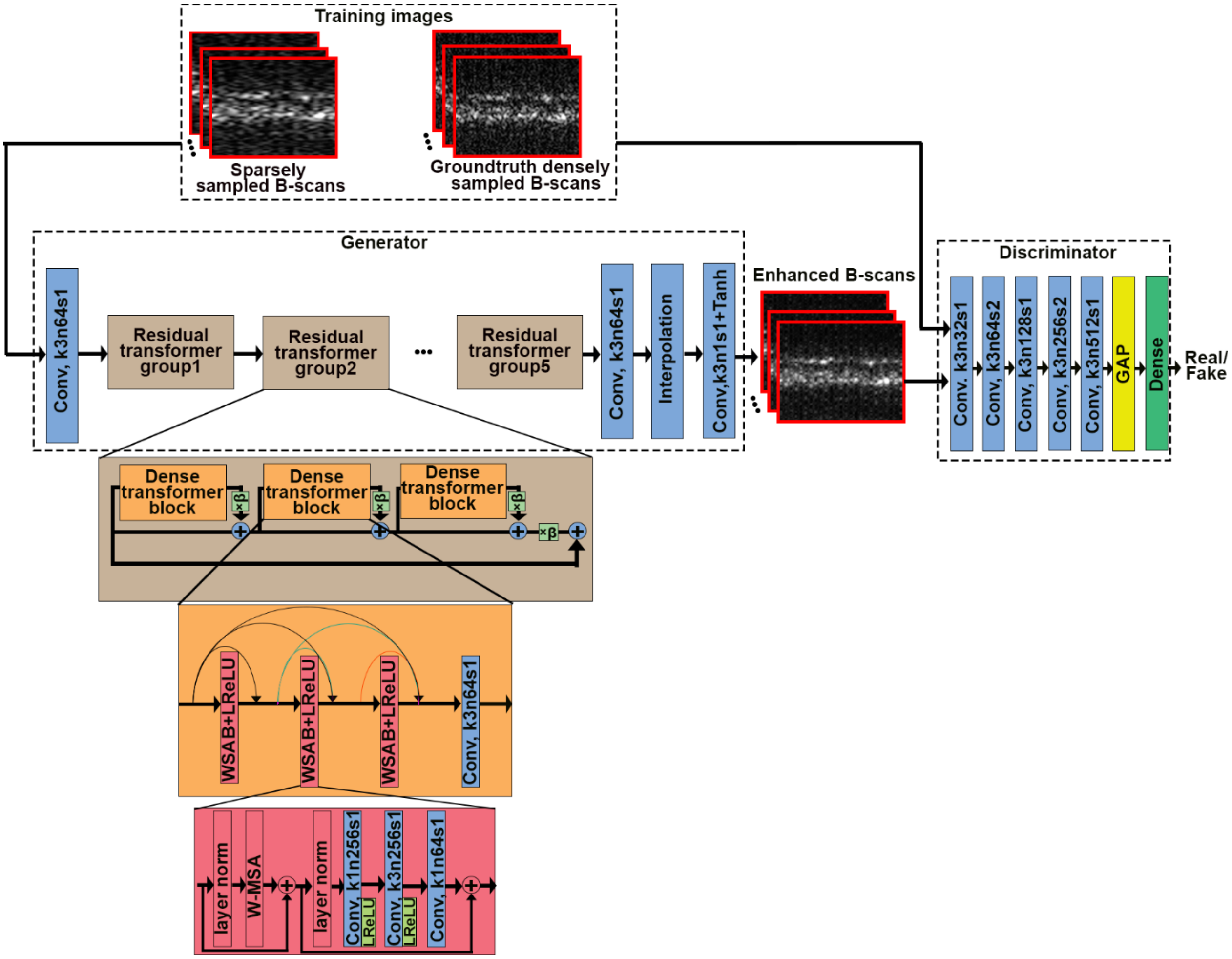

Supplementary Fig. 1. Network architecture of residual in residual transformer generative adversarial network (RRTGAN). The generator (G) takes the input sparsely sampled B-scans and enhances the images using a series of convolution and transformer blocks. The generator first employs a convolution layer (Conv) to extract spatial features from the input images. These features are then provided to a series of five residual transformer groups to extract non-local correlations in the images. These features are then summarized using another convolutional layer and restored to the original spatial size by interpolation. In the end, a convolutional layer with Tanh activation is used to obtain the enhanced images. k, n, and s in the convolutional layers denote kernel size, number of filters, and stride of the convolution. The residual transformer groups consist of multi-level residual network of dense transformer blocks. Each of the dense transformer blocks have three densely connected windowed self-attention blocks (WSABs) and a convolution layer. Each of the WSABs are followed by the rectified linear unit (ReLU) activation function. The WSAB starts with a normalization layer (layer norm) followed by windowed multi-head attention (W-MSA) module that divides the feature maps into non-overlapping patches and computes the self-attention for each of the patches. The output of each window is then merged to obtain the output feature map. The W-MSA is followed by layer normalization and three convolutional layers. The discriminator takes as input the ground truth densely sampled B-scans and the images enhanced by the generator to assess the visual and perceptual similarity between them by assigning labels of fake/real based on the similarity between the images. The discriminator has a series of convolutional layers, followed by global average pooling (GAP) layer and dense layer.

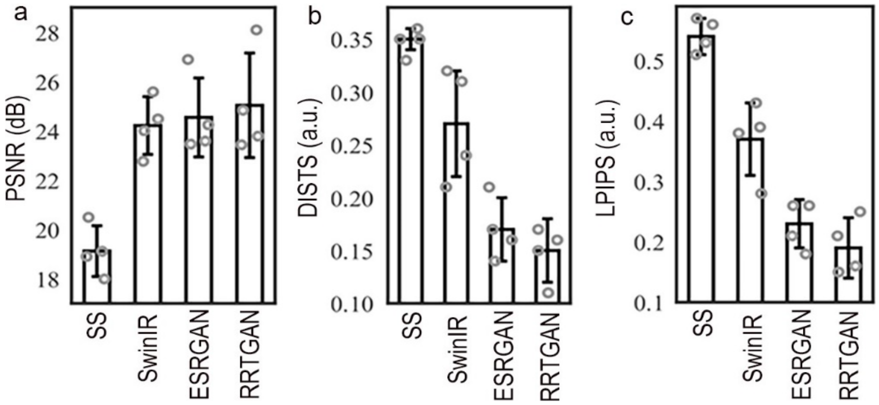

Supplementary Fig. 2. Residual in residual transformer generative adversarial network (RRTGAN) consistently outperforms other methods across different participants. The bar graphs indicate the average performance of the sparsely sampled (SS) images and the artificial intelligence (AI) frameworks across four participants (shown in circles) compared using **(a)** peak signal to noise ratio (PSNR), **(b)** deep image structure and texture similarity (DISTS), and **(c)** learned perceptual image patch similarity (LPIPS). Error bars denote standard deviation.

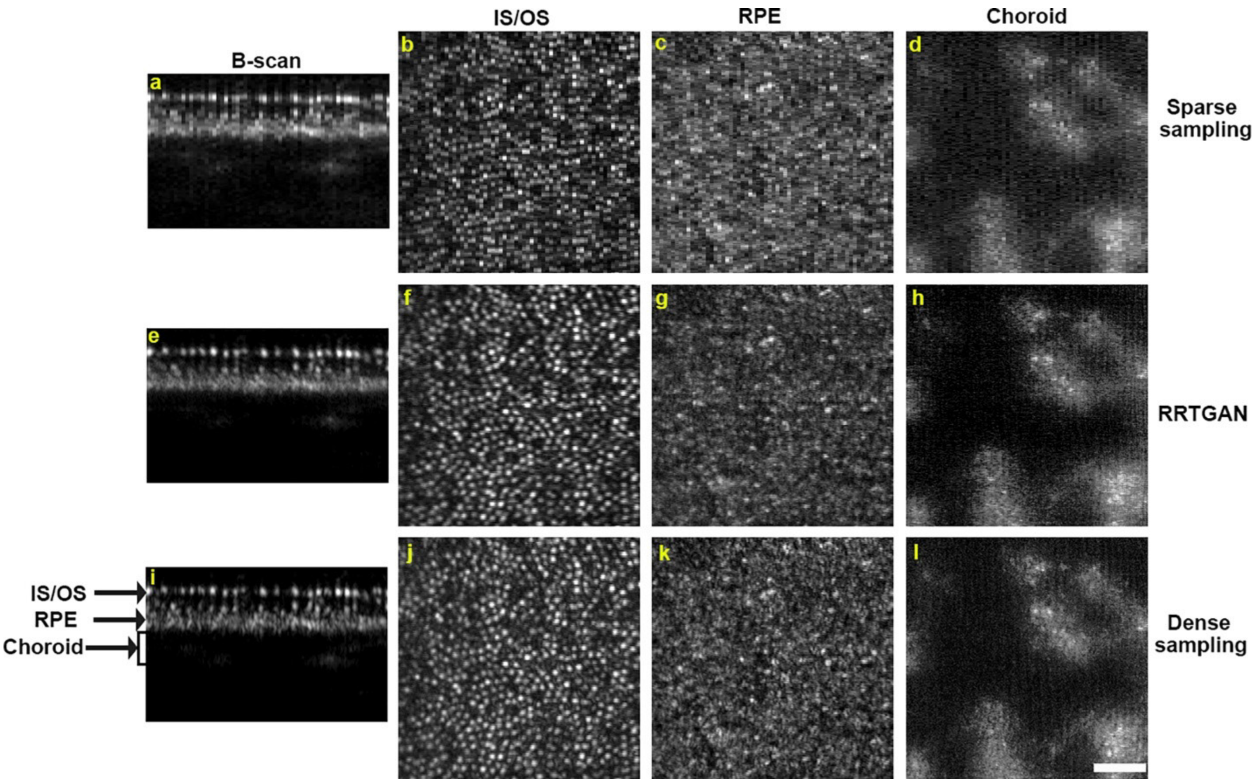

Supplementary Fig. 3. Residual in residual transformer generative adversarial network (RRTGAN) restores cells from deeper layers of the retina beneath cones. (a, e, i) Cross-sectional adaptive optics optical coherence tomography (AOOCT) B-scans of the sparsely sampled, RRTGAN enhanced and ground truth densely sampled retina of participant P1 at 2.5 mm temporal to the fovea. Top-down view of the photoreceptor **(b, f, j)** inner segment/outer segment (IS/OS) junction, shown for reference, alongside **(c, g, k)** retinal pigment epithelial (RPE) cells and **(d, h, l)** choroidal vessels for the sparsely sampled, RRTGAN and densely sampled images**.** Bright dots in (**b, f and j**) are individual cone photoreceptors. Dark spots in (**c, g, and k**) are the centers of individual RPE cells. The images of RPE cells shown here are extracted from a single non-averaged AOOCT volume, affected by speckle noise due to the lack of averaging across time. The round/oval patches in (**d, h, l**) are choroidal vessels. Scale bar: 50 μm.

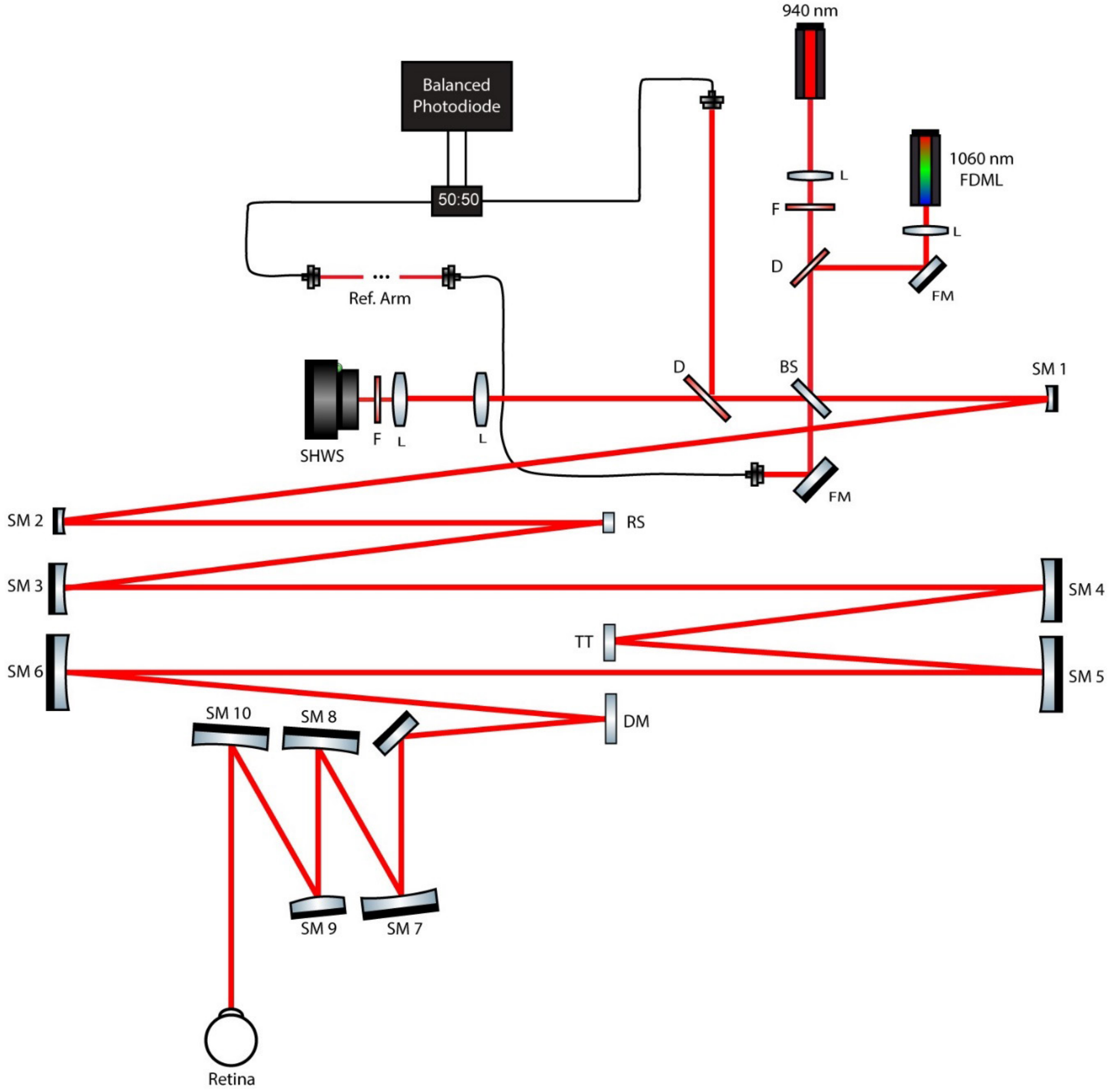

Supplementary Fig. 4. Schematic of the custom swept source AOOCT instrument. A 3 MHz 1060 nm Fourier domain mode-locked laser (FDML) for OCT is combined with a 940 nm wavefront sensor (WFS) beacon for AO. The sample and reference arms are separated by a 90/10 beam splitter (BS). The light returning from the retina is descanned and transmitted through the BS where it is reflects off a dichroic mirror (D), and then is interfered with light from the reference arm to form the OCT image. Abbreviations: BS- beam splitter, D- dichroic mirror, DM- deformable mirror, F- clean up filter, RS- resonant scanner, SHWS- Shack-Hartmann wavefront sensor, SM- spherical mirror, L- lens, FM- fold mirror, and TT- tip/tilt scanner.

Supplementary Tables

Supplementary Table 1. Participant information

ID	Age	Eye †	Locations imaged*	Participant data used for training and validating AI model	Locations used for AI training	External validation††
P1	55-59	OD	0-2.8 mm, step size of 0.1 mm	No	N/A	Yes
P2	45-49	OS	0.6, 0.8, 1.1, and 1.4 mm	Yes	0.8 mm	No
P3	30-34	OD	0.3,0.8,1.3, 1.9, and 2.4 mm	Yes	0.8 mm	No
P4	30-34	OD	0.6,0.9,1.2, and 1.4 mm	Yes	0.9 mm, 1.2mm	No

†OD- right eye, OS- left eye

*All locations are imaged temporal to the fovea. At each location, AOOCT volumes are acquired using a field of view of 1.5 degrees.

†† This data is never seen by the AI model during training and is used to demonstrate the generalizability of the AI model and large-scale visualization of cone photoreceptors across the retina.

Supplementary Table 2. Comparison of edge variances across four participants

Image	Edge variance† (mean ± SD)
Sparsely sampled	28.0 ± 19.1
ESRGAN	2.2 ± 0.7
SwinIR	1.8 ± 0.5
RRTGAN (ours)	1.9 ± 0.3

†The edge variance values are normalized with respect to the edge variance of the densely sampled images.

Supplementary Table 3. Cone photoreceptor cell spacing of four participants estimated from the power spectrum analysis

Participant	Distance from fovea (mm)	Cell spacing (μm)				
		Sparse sampling	ESRGAN	SwinIR	RRTGAN (ours)	Dense sampling
P1	1.13	8.1	8.1	8.1	8.1	8.1
P2	1.10	9.3	9.0	9.0	9.0	9.0
P3	1.34	9.4	8.9	8.9	8.9	8.9
P4	1.16	8.3	8.3	8.3	8.3	8.3

Supplementary Table 4. Enhancement performance of RRTGAN at four locations that are never seen during training for participant P1

Distance from fovea (mm)	DISTS (a. u.)	PSNR (dB)	LPIPS (a. u.)
0.8	0.14	26.34	0.17
1.4	0.09	27.80	0.10
1.8	0.07	30.20	0.08
2.5	0.10	30.10	0.13

Supplementary Table 5. Network characteristics and times

**Characteristics**	**ESRGAN**	**SwinIR**	**RRTGAN (ours)**
No. of parameters	9.8	3.1	5.2
Training image size	Input: 40 × 100<br>Ground truth: 40 × 400	Input: 40 × 100<br>Ground truth: 40 × 400	Input: 40 × 100<br>Ground truth: 40 × 400
Number of paired training images	10,232	10,232	10,232
Batch size	32	32	32
Number of epochs	50	50	50
Learning rate	0.0002	0.0002	0.0002
Optimizer	Adam	Adam	Adam

The AI models were trained and tested using 4 NVIDIA A100 GPUs.

Supplementary video captions

Supplementary Video 1: **RRTGAN restores the pixel sampling of cones from sparsely sampled AOOCT.** Toggling between sparsely sampled and RRTGAN enhanced images for participant P3 shows that RRTGAN has restored the pixel resolution and circular shape of the individual cone cells. RRTGAN shows visual similarities with the densely sampled ground truth image (right). Scale bar: 50 μm.

Supplementary Video 2: **RRTGAN preserves the spatial locations of the cones in acquired images at different times.** Toggling between RRTGAN enhanced images acquired at two overlapping retinal locations for participant P1 shows that the cone cells restored by RRTGAN are consistent in the overlapping regions. A small amount of distortion arising from the combination of continuous eye motion with scanner motion is present, common to all point-scanning AO systems. The yellow arrows highlight a few cones that appear in both the images. Scale bar: 100 μm.

Supplementary References

1. Goodfellow, I., et al., Generative adversarial nets. Advances in neural information processing systems, 2014. 27.
2. Wang, X., et al. Esrgan: Enhanced super-resolution generative adversarial networks. in Proceedings of the European conference on computer vision (ECCV) workshops. 2018.
3. Ledig, C., et al. Photo-realistic single image super-resolution using a generative adversarial network. in Proceedings of the IEEE conference on computer vision and pattern recognition. 2017.
4. Das, V., et al., Revealing speckle obscured living human retinal cells with artificial intelligence assisted adaptive optics optical coherence tomography. Communications Medicine, 2024. 4(1): p. 68.
5. Yang, Q., et al., Low-dose CT image denoising using a generative adversarial network with Wasserstein distance and perceptual loss. IEEE transactions on medical imaging, 2018. 37(6): p. 1348-1357.
6. Xie, Z., et al., Generative adversarial network based regularized image reconstruction for PET. Physics in Medicine & Biology, 2020. 65(12): p. 125016.
7. Lv, J., et al., Transfer learning enhanced generative adversarial networks for multi-channel MRI reconstruction. Computers in Biology and Medicine, 2021. 134: p. 104504.
8. Liu, Z., et al. Swin transformer: Hierarchical vision transformer using shifted windows. in Proceedings of the IEEE/CVF international conference on computer vision. 2021.
9. Liang, J., et al. Swinir: Image restoration using swin transformer. in Proceedings of the IEEE/CVF international conference on computer vision. 2021.
10. Ding, K., et al., Image quality assessment: Unifying structure and texture similarity. IEEE transactions on pattern analysis and machine intelligence, 2020. 44(5): p. 2567-2581.
11. Zhang, R., et al. The unreasonable effectiveness of deep features as a perceptual metric. in Proceedings of the IEEE conference on computer vision and pattern recognition. 2018.
12. Heusel, M., et al., Gans trained by a two time-scale update rule converge to a local nash equilibrium. Advances in neural information processing systems, 2017. 30.
13. Cooper, R.F., G.K. Aguirre, and J.I. Morgan, Fully automated estimation of spacing and density for retinal mosaics. Translational vision science & technology, 2019. 8(5): p. 26-26.
14. Bennett, A. and R. Rabbetts, Proposals for new reduced and schematic eyes. Ophthalmic & Physiological Optics: the Journal of the British College of Ophthalmic Opticians (Optometrists), 1989. 9(2): p. 228-230.
15. Liu, J., et al., Automated photoreceptor cell identification on nonconfocal adaptive optics images using multiscale circular voting. Investigative ophthalmology & visual science, 2017. 58(11): p. 4477-4489.
